# The innovative and purpose-built veterinary antimicrobials sales reporting system

**DOI:** 10.14745/ccdr.v51i09a04

**Published:** 2025-10-09

**Authors:** Shamir Mukhi, Manisha Mehrotra, Carolee A Carson, Xian-Zhi Li, Mark Reist, Angelina L Bosman, Annika Flint, Valentine Usongo, Ben Gammon, Tim Beattie

**Affiliations:** 1 Canadian Network for Public Health Intelligence, Public Health Agency of Canada; 2 Veterinary Drugs Directorate, Health Canada, Ottawa, ON; 3 Centre for Food-borne, Environmental and Zoonotic Infectious Diseases, Public Health Agency of Canada, Guelph, ON

**Keywords:** VASR, Canada, veterinary antimicrobials, sales, antimicrobial use, antimicrobial resistance, animals

## Abstract

**Background:**

Antimicrobial resistance (AMR) is one of the major public health threats of our time. Human activities across the One Health spectrum, such as the misuse and overuse of antimicrobials, can accelerate the resistance threat. A variety of antimicrobials used in veterinary medicine are also important in human medicine. As part of Canada’s commitment to address AMR and antimicrobial use (AMU), and to align with international best practices aimed to minimize the impacts of AMR and preserve the effectiveness of existing antimicrobials, regulatory controls and enhanced surveillance initiatives have been implemented in veterinary medicine and animal health to improve intelligence on the quantities of antimicrobials available for use in animals. These efforts include the implementation of the national Veterinary Antimicrobial Sales Reporting (VASR) system in Canada, in 2018. The focus of this article is to describe the VASR data collection system and platform.

**Methods:**

A custom-built data collection and analytical system was developed to enhance understanding of the volume of antimicrobials available for use in animals and contribute to the broader surveillance of trends in AMU and AMR in an effort to support stewardship. Partners from Health Canada’s Veterinary Drugs Directorate, the Public Health Agency of Canada’s Centre for Food-borne, Environmental and Zoonotic Infectious Diseases, and the Canadian Network for Public Health Intelligence worked together to envision, develop, and implement the national-scale, purpose-built technological intervention, the VASR system.

**Results:**

The VASR surveillance system provides a robust data collection and analytical informatics platform to improve intelligence on antimicrobial sales available for veterinary use in Canada.

**Conclusion:**

An innovative, purpose-built national antimicrobial sales reporting system was developed. This web-based platform is effective for data submission by the participants and facilitates analysis to provide a comprehensive picture of medically important antimicrobials available for use in animals in Canada, thereby supporting AMR and AMU surveillance and stewardship.

## Introduction

The World Health Organization (WHO) continues to stress the need for action, characterizing antimicrobial resistance (AMR) as one of the most pressing public health and development threats of our time (1). Antimicrobials include antibiotics, antibacterials, antivirals, and antifungals used to treat infectious diseases in humans, animals and plants/crops. Antimicrobial resistance occurs naturally over time as microbes adapt and become resistant to antimicrobials to which they were exposed. While AMR makes diseases more difficult to treat, it can also increase the risks of disease transmission and severe outcomes (2). Anytime antimicrobials are used, resistance can develop and spread, and the overuse and misuse of antimicrobials is a key driver of this process (3).

To address the complex challenge posed by AMR, the WHO has advocated since 2005 that actions are required within the areas of human health, food production, animal and environmental health, and that such efforts need to be coordinated both within national action plans as well as internationally, through a global One Health strategy ((3)). In 2014, various countries, including Canada, endorsed a World Health Assembly resolution committing to the development of national action plans and international coordination of efforts to counter the risks posed by AMR (4). In 2015, the World Organisation for Animal Health (WOAH)—formerly the Office International des Epizooties (OIE)—began collecting data on antimicrobials intended for use in animals, as part of the global response to AMR. In 2023, the Pan-Canadian Action Plan on AMR established a five-year (2023−2027) blueprint to coordinate and accelerate the national response to address AMR and antimicrobial use (AMU) ((5)).

In the spring of 2015, the Office of the Auditor General of Canada published a report on AMR, which identified that more work was needed to advance the national strategy and improve surveillance ((6)). This included the need for further actions in support of the prudent use of antimicrobials. Regulatory gaps were identified with respect to the oversight of veterinary drugs, including antimicrobial drugs of importance in human medicine ((6)). In response, Health Canada implemented a number of regulatory and policy changes in 2017 and 2018, and addressed a number of regulatory gaps to improve oversight and strengthen the responsible use of antimicrobials in animals. These regulatory changes included new rules for importation and quality of active pharmaceutical ingredients for veterinary use, restrictions on personal importation of drugs for food–producing animals, and the introduction of a new pathway for veterinary health products. The changes to the Food and Drug Regulations also established the mandatory requirement for antimicrobial sales reporting (7). Any antimicrobial drugs included in Health Canada’s List A (certain antimicrobial active pharmaceutical ingredients that are important in human medicine) ((8)), intended for use in animals, are subject to annual sales reporting. These regulations also require data providers to estimate their sales of medically important antimicrobials by different animal species groups.

The focus of this paper is to recount the collaborative work leading to the creation of the innovative Veterinary Antimicrobial Sales Reporting (VASR) system, an adaptable data collection and analytical informatics solution designed and purpose-built by the Canadian Network for Public Health Intelligence (CNPHI) team at the Public Health Agency of Canada (PHAC), in collaboration with program experts.

## Methods

### Development of the Veterinary Antimicrobial Sales Reporting platform

Between 2005 and 2018, the Canadian Animal Health Institute (CAHI), a trade organization representing the animal health market in Canada ((9)), voluntarily provided data on the quantities of antimicrobials distributed for sale in animals from their members to PHAC’s Canadian Integrated Program for Antimicrobial Resistance Surveillance (CIPARS) ((10). Each year, CAHI members covered 90%–95% of the animal health market, with the data stratified by province/territory and animal type (i.e., either production or companion animals). With the regulations coming into effect in November 2017, a commitment was set to establish 2018 as the first year for mandatory reporting of medically important veterinary antimicrobial sales from those who manufacture, import or compound, with the deliverable to be accomplished and data reports submitted by March 31, 2019.

Building on the voluntary data and data structure provided by CAHI, CIPARS worked with counterparts in the European Union (European Medicines Agency) ((11)) and the United States (Food and Drug Administration) ((12)) to review their data collection and management tools (including lessons learned) for sales data. Informal consultations were also held with counterparts in Japan. CIPARS also gained experience from participating in the development of WOAH’s global database on antimicrobial agents intended for use in animals ((13)), which later became the ANImal antiMicrobial USE (ANIMUSE) global database (14). However, no tools were developed to collect data across more than ten different animal species while incorporating regional stratification. Hence, the VASR system needed to provide a modified and novel approach.

The objective of the initiative was to conceptualize, design, develop, test and launch a robust data collection and analytical informatics solution to enable a national-scale surveillance capability, yielding improved intelligence on antimicrobial sales for veterinary use in Canada. Partners from Health Canada’s Veterinary Drugs Directorate (VDD) and the Food-borne Disease and Antimicrobial Resistance Surveillance Division (FDASD) at PHAC met with CNPHI for the first time in December 2016. Numerous planning and design meetings followed throughout 2017 to focus on the functionality and effectiveness of a data collection and analysis system. In keeping with their philosophy of working closely with public health program partners to fully understand their needs and vision, CNPHI worked with program experts to conceptualize the interoperable components that would comprise the VASR system. This would require versatility to accommodate the nuances and detailed complexities of the data and the required preliminary analyses. As a result, the VASR system was purpose-built to consist of a suite of interoperable systems within a single platform to achieve the required outcomes. The VASR platform components include:

• **Participant management:** Functionality is required to securely manage the private access of numerous data providers and categorize their roles as manufacturers, importers or compounders, as well as their contact and notification details. Over time, this component is designed to enable participant-specific summary reports, fully characterizing the products and formulations of veterinary antimicrobial products sold.

• **A user reporting portal:** The reporting portal provides a secure and easy-to-use means for data providers to submit reports directly into the VASR system.

• **Code management:** This customized component is required for managing hierarchical classifications of veterinary medicines using the international Anatomical Therapeutic Chemical classification system for veterinary medicinal products (ATCvet) (5^th^ level), which facilitates the exchange and comparison of data on veterinary drug use at international and national levels. The code management functionality offers adaptability to accommodate periodic changes in substance naming and coding standards ((15)).

• **Product management and unit management:** An agile functionality is designed for managing and categorizing products, capturing drug identification numbers (DINs), product names, active ingredients, drug class, categories based on importance in human medicine, and product use (e.g., preventive, therapeutic), and providing the capability to link products to the ATCvet coding system. Furthermore, the system is pre-populated with information on over 400 Health Canada authorized veterinary antimicrobial products (that have DINs), streamlining the ease of sales reporting by participants.

Product formulations can vary widely, impacting how participants report sales. For example, formulations may include powders, tablets or vials, and quantities may be expressed in varying units, such as the number of packages, bulk mass or volume. As a result, the system offers tools to convert results to be consistently presented in kilograms sold.

In addition, some products reported may be prodrugs. A prodrug acts as a precursor (parent compound), which in turn undergoes a metabolic transformation once administered into the body of the animal, resulting in the presence of the active ingredient ((16)). Participants report the quantity of prodrugs sold in terms of the quantity of parent compound, which requires the application of a prodrug conversion factor to accurately reflect the quantity of active ingredient reported ((17)).

The practice of compounding also introduces complexities in data collection, reporting, and analysis. A compounded drug can be defined as an approved drug that has been manipulated to achieve a dosage, form or concentration other than that specified on the label; the combining of two or more drugs; a dilution of a drug other than that prescribed on the label; or the creation of a mixture to be administered by a different route. Compounding is an acceptable practice in veterinary medicine when no authorized product or formulation is available, typically carried out by a pharmacist or veterinarian ((18)) to fulfill an unmet need for a client. However, this practice bypasses federal pre–market authorization and safety reviews.

The main goal of the product management component is to consistently support surveillance of how much product is sold by product type, unit/size, DIN, formulation, an estimate of the product sales by animal/animal species group, distribution of package sales by province/territory, active ingredients, and total kilograms sold nationally and by province/territory. Animal species are specified to the categories of aquaculture, cattle (beef), cattle (dairy), cattle (veal), chickens, companion animals, horses, pigs, small ruminants, turkeys, and “others”. While reporting of estimated antimicrobial sales by animal species is mandatory as per regulatory requirements, the information on provincial/territorial distribution is encouraged by the VASR administrator (i.e., VDD), in an effort to obtain relevant and robust information from the data providers. This includes:

• **Notification management and reporting compliance:** The secure notification management component facilitates the management of participant notifications and reporting reminders to assist the program in overseeing reporting compliance and validation.

• **Data collection:** To adequately support VASR’s objectives, a custom-designed system for data flow is required. The data collection system enables the capture of details on each product sold, providing the capability to delineate sales by attributes of analytical and importance to human medicine. Data collection is initiated in conjunction with the notification system, whereby participants are made aware that reporting is due and are provided with a secure, dedicated link to access the online reporting. For ease of use, the system allows participants to save their data collection forms as drafts until finalized for submission. The flexibility and adaptability of the data collection system is key, as this allows participants to report sales according to their particular method of product packaging and labelling.

• **Review and validation of data submissions:** In the event that errors are detected during the program’s review process, a resubmission capability is integrated within the process, leveraging the notification system to prompt participants to review and correct a submission, when required. For overdue reports, a built–in ‘days overdue’ indicator assists VDD program staff in identifying overdue reports and initiating reminders or communicating other required compliance actions to participants through the notification system. A customized aberration detection system is designed to flag potential anomalies in the data submitted for each product within a given submission, using historical data, supporting proactive validation and quality assurance.

• **Analytics:** Purpose-built analytical tools are designed to yield optimal intelligence from the collected data, providing readily available preliminary charts, trends and visualizations. From an interactive dashboard, the statuses of incoming reports are summarized to facilitate the ongoing tracking of submissions from participants, flagging reports as draft, submitted, accepted, and completed. It also flags reports containing potential errors and those noting no data to report. Reports are categorized according to their source, originating from manufacturers, importers and compounders, as well as the year the reports were submitted and their status.

As data on veterinary drug sales accumulates, the analytical features provide the capability to produce preliminary visual trends, insights and comparisons over time, including:

• Total drug sales reported, delineated by drug class, nationally or by province/territory, with estimates of percentages of totals by animal species/animal species groups

• Sales of antimicrobials delineated by their importance in human medicine, drug use purposes (e.g., treatment, prevention), antimicrobial classification, animal species and route of administration

• Visualization of annual percentage changes in the use of medically important drugs by antimicrobial classification

• Product summaries describing product names, company names, active ingredients, DINs and species

• Various unit conversion tools to support consistent surveillance results in kilograms sold from reported quantities of prodrugs and compounded drugs, as well as reports using varied product strengths or number of packages sold

The system also provides insights and resources to support the application of ATCvet groups and codes, and to fully describe products according to their related active ingredients, DINs, product name, category, company, and species.

## Results

As the major outcome, the web-based VASR system was successfully developed and launched, with the first collection of veterinary antimicrobial sales data in the system for the year 2018. While voluntary reporting had been supported in previous years by CAHI, the newly established system provides innovations that resulted in various improvements in the sales reporting for this first year of mandatory collection ((19)). The VASR participants (i.e., data providers from manufacturers, importers and compounders) can enter and save the required product-by-product information in the system before submitting their final reports. For each participant, a sales summary report is generated, containing all required information, enabling the timely review of reported product information by VASR administrators to identify any missing or incorrect information. When issues are identified, participants are contacted to verify or correct submitted information. This timely review of submissions and communication between the VASR administrators and participants plays a key role in ensuring the data quality.

Since data reporting became mandatory, more data providers have participated in comparison to previous years of voluntary reporting by CAHI members, including sales data per animal species/groups and data from importers and compounders. New data were collected on sales in the territories, resulting in more nationally comprehensive data ((20)).

As the VASR system is a custom-built reporting interface through the CNPHI platform, updates can be made to the interactive forms based on user-experience and feedback to optimize data entry for providers and enhance reporting completeness and accuracy. Following each reporting year, the adaptability of the system allows for improvements that increase the ease of use for participants by supporting the ability to update and revise the previous year’s submission, save draft reports in progress, and to submit a ‘no sales to declare’ response when applicable. Overall, the functionality of the system has improved over the last seven years, based on the experience from VASR participants and administrators.

As familiarity with the system has grown, voluntary reporting of sales by animal species at the provincial/territorial level has increased. Improvements in reporting of sales for minor species (e.g., small ruminants or “other species”) have also been noted. Comparisons of reported sales for use in aquaculture with use data from aquaculture operations mandatorily reported to Fisheries and Oceans Canada ((21)) show very similar results between the two information sources, highlighting sales as a reliable indicator for use.

To date, annual sales reporting through the VASR system has been completed for six years (2018−2023), and reporting for the seventh year (2024) is currently underway. These data collection, analysis and reporting capabilities have achieved program objectives, resulting in the timely release of the data to the public via an interactive visualization ((22)).

## Discussion

The VASR system provides an innovative, purpose-built platform with functional versatility. The system enables effective reporting of antimicrobials sales for veterinary use from the participants while also supporting the timely validation of submissions and data quality by VASR administrators, with the flexibility to modify components of the data capture system based on real-word experience and user feedback. Despite the complexity of the data, annual data collection, analysis and public reporting can be completed within months ((22)).

Overlapping reporting of antimicrobial sales in 2018 during the transition from data collection through CAHI and VASR provided an opportunity to compare the coverage between the two datasets. The quantity of sales (in kilograms) reported through VASR by manufacturers and importers in 2018 were 1.12 times higher than those reported through CAHI (by CAHI member manufacturers only) that same year. This reflects the importance of both the regulatory changes (mandatory data reporting) and the VASR system in achieving data capture beyond that achieved voluntarily through CAHI. To examine trends in antimicrobial sales over time for periods before 2018, the CAHI data were multiplied by 1.12 to adjust for the difference in coverage between the two sources of data, which enabled the historical comparison of decades-long veterinary antimicrobial sales data in Canada.

The creation of the VASR system not only allowed participants to fulfill the regulatory requirement of reporting sales information, it also helps governmental program staff to monitor the patterns of antimicrobials intended for use in animals in Canada. The latter are further analyzed by CIPARS in conjunction with the findings from ongoing active and passive surveillance of AMR and AMU in food animals and their derived food products ((23)). Together, these complementary aspects are essential for integrated surveillance under the One Health spectrum.

The system allows for, and encourages, the optional reporting of provincial/territorial sales distribution information. Given the importance of sales distribution across provinces and territories, the VASR administrators and participants have made efforts to work together to report provincial and territorial sales data as evident in recent sales reporting ((22)), which was largely attributed to the user-friendly functionality of the system and allowed for streamlined communications.

The easily produced and readily available extracts of VASR data in a csv format have facilitated deeper data validation processes and analytics using other software, activities that are beyond the scope of traditional database analytics and which require veterinary and epidemiological expertise. The resulting data have been presented in the annual CIPARS stakeholder meeting held every November during World Antimicrobial Resistance Awareness Week. The data are also summarized through VASR highlights reports. There are currently six published reports, and the data are available publicly via interactive data ((22)). Importantly, data from VASR have been provided annually as Canada’s submission to the ANIMUSE database on antimicrobial agents intended for use in animals ((14,17)).

## Limitations

There are limitations due to challenges with accurate provincial-level species reporting, as all data providers may not have these details available. This can lead to potential gaps and an incomplete picture of sales trends by species at the provincial level. These data are often requested by stakeholders. To ensure the data are as complete as possible, the VASR administrators regularly engage with the data providers to encourage the submission of this information as best estimates, in order to enhance data quality and species-level insights to inform targeted AMR and AMU stewardship and surveillance. While the completeness of the provincial-level species data may vary between species, in 2023, 92% of the total antimicrobial sales reported nationally at the species level were also reported provincially (an increase from 25% in 2018).

In addition, the VASR system relies on annual submissions from data providers across Canada. Despite the reporting requirement, compliance challenges remain in ensuring comprehensive awareness of the duty to report sales, especially among individuals and companies compounding the implicated products. To address this, concerted efforts are made via targeted emails, bulletins, and postings to inform all potential data providers and provincial authorities about VASR, its significance, and the annual reporting obligation, encouraging good compliance year to year.

## Conclusion

The VASR system is an efficient, highly functional and sustainable system. Its design-for-purpose helps facilitate validation and deeper analysis of the data by Health Canada and PHAC analysts and veterinary epidemiologists. Experience gained from the last six years of annual reporting will help to further enhance the functionality of the system. The information generated from the system is indispensable in achieving an enhanced understanding of antimicrobial sales patterns and trends, and to support antimicrobial stewardship and further surveillance.
